# Development and validation of the low‐cost pregnant female physical phantom for fetal dosimetry in MV photon radiotherapy

**DOI:** 10.1002/acm2.14240

**Published:** 2023-12-27

**Authors:** Vjekoslav Kopačin, Hrvoje Brkić, Ana Ivković, Mladen Kasabašić, Željka Knežević, Marija Majer, Marijana Nodilo, Tajana Turk, Dario Faj

**Affiliations:** ^1^ Faculty of Medicine Department of Radiology J. J. Strossmayer University Osijek, University Hospital Center Osijek Department of Diagnostic and Interventional Radiology Osijek Croatia; ^2^ Faculty of Medicine Department of Biophysics and Medical Physics J. J. Strossmayer University Osijek Osijek Croatia; ^3^ Faculty of Dental Medicine and Health Department of Biophysics Biology and Chemistry J. J. Strossmayer University Osijek Osijek Croatia; ^4^ Faculty of Medicine Department of Biophysics and Medical Physics J. J. Strossmayer University Osijek, University Hospital Center Osijek Department of Medical Physics Osijek Croatia; ^5^ Division of Materials Chemistry Ruđer Bošković Institute Zagreb Croatia

**Keywords:** breast neoplasm, fetus, megavolt radiotherapy, phantom, physical, pregnant, radiation dosimetry, woman

## Abstract

**Background:**

Monte Carlo (MC) simulations or measurements in anthropomorphic phantoms are recommended for estimating fetal dose in pregnant patients in radiotherapy. Among the many existing phantoms, there is no commercially available physical phantom representing the entire pregnant woman.

**Purpose:**

In this study, the development of a low‐cost, physical pregnant female phantom was demonstrated using commercially available materials. This phantom is based on the previously published computational phantom.

**Methods:**

Three tissue substitution materials (soft tissue, lung and bone tissue substitution) were developed. To verify Tena's substitution tissue materials, their radiation properties were assessed and compared to ICRP and ICRU materials using MC simulations in MV radiotherapy beams. Validation of the physical phantom was performed by comparing fetal doses obtained by measurements in the phantom with fetal doses obtained by MC simulations in computational phantom, during an MV photon breast radiotherapy treatment.

**Results:**

Materials used for building Tena phantom are matched to ICRU materials using physical density, radiation absorption properties and effective atomic number. MC simulations showed that percentage depth doses of Tena and ICRU material comply within 5% for soft and lung tissue, up to 25 cm depth. In the bone tissue, the discrepancy is higher, but again within 5% up to the depth of 5 cm. When the phantom was used for fetal dose measurements in MV photon breast radiotherapy, measured fetal doses complied with fetal doses calculated using MC simulation within 15%.

**Conclusions:**

Physical anthropomorphic phantom of pregnant patient can be manufactured using commercial materials and with low expenses. The files needed for 3D printing are now freely available. This enables further studies and comparison of numerical and physical experiments in diagnostic radiology or radiotherapy.

## 1 INTRODUCTION

The use of therapeutic procedures and diagnostic methods based on ionizing radiation during pregnancy is still something of a taboo for many clinicians and radiologists, and opinions may differ considerably. Although there are recommendations such as those of the ICRP^1^ and FIGO^2^ that ionizing radiation can be used in pregnant women if the dose does not exceed 50–100 mGy, many professional societies explicitly prohibit its use.^3^, ^4^, ^5^, ^6^, ^7^, ^8^, ^9^, ^10^, ^11^, ^12^, ^13^

Research on pregnant women would be unethical and inhumane, therefore it is necessary to conduct experiments on phantoms, either computerized or physical. Recommended methods for estimating the fetal dose in pregnant patients include detailed Monte Carlo (MC) simulations or measurements in anthropomorphic phantoms.^14^, ^15^

According to the “Handbook of Anatomical Models for Radiation Dosimetry,”^16^ since the 1960s among the 121 phantoms, only a few pregnant woman phantoms were developed: computerized pregnant woman phantoms RPI‐P3, RPI‐P6, RPI‐P9,^17^⁠ followed by Katja,^18^ the Japanese Atomic Energy Agency pregnant woman phantom^19^ and the UF family pregnant woman.^20^ The mentioned computer phantoms were created by simply “inserting” the fetus model, obtained by subsequent scanning of other pregnant women using magnetic resonance imaging (MRI), into previously developed female phantoms. The fetal anatomy was additionally modeled according to the physiognomy of the female phantom, which was also adapted to the fetus model. Because of their prices, computational phantoms are often unavailable to organizations and groups of scientists who are engaged in research in the field of dosimetry of pregnant women and fetuses,^17^ while a commercial physical phantom of a pregnant woman still does not exist.

According to the literature search, only a few non‐commercial physical phantoms of a pregnant woman have been made so far. The first is a modular phantom of the abdomen and pelvis of a pregnant woman in the 15^th^, 25^th^, and 38^th^ week of pregnancy, which is based on a computerized phantom of a pregnant woman from the UF phantom family,^21^ next is a phantom manufactured at Kermanshah University of Medical Sciences in Iran representing a pregnant woman of the undetermined duration of pregnancy​,^22^ then a phantom for experimental measurement of fetal radiation during diagnostic CT examination of a pregnant woman's abdomen​^23^​ and a similar one made by Hurwitz et al.^24^

This study demonstrated the development of a low‐cost, physical pregnant female phantom using commercially available materials. This phantom is based on the previously published computational phantom, developed by our group.^25^ Though MC simulations are valuable research methods, their setup and computational time can take a significant amount of time.^26^, ^27^ In addition, MC simulations are subject to uncertainties such as underestimation of the absorbed dose outside the primary radiation field.^28^, ^29^, ^30^, ^31^, ^32^ Therefore, physical in‐phantom measurements are indispensable and allow an experimental measurement of the fetal dose enabling validation of MC simulations. Moreover, in the clinical environment, physical measurements are often required for such pregnancy cases and further allow establishing protocols for estimating dose conversion coefficients for in vivo dosimetry.^33^, ^34^ For those reasons, we decided to make the physical representation of the Tena phantom since we already have prepared both voxelized forms and .STL (eng. “Standard Tessellation Language”, stereolithography) files suitable for 3D printing of the phantom, in the previously published study.^25^ As proof of principle, we performed experimental validation of the physical phantom comparing experimentally determined fetal doses, using radio photoluminescence (RPL) dosimeters, with simulations during a breast radiotherapy treatment of 50 Gy.

## 2 MATERIALS AND METHODS

As described previously,^25^ a computational pregnant female phantom named Tena was developed based on a female patient of central European descent in the 18^th^ week of pregnancy.

### 2.1 Physical phantom development

#### 2.1.1 3D modeling and 3D printing of the molds

Since the computational phantom has 31 structures available in the STL format, they were imported into the “open‐source” 3D modeling software Blender,^35^ where 3D models of molds for the tissue substitute material casting were generated. The models of organs and structures served as a landmark for placing the cylinders in space measuring 4.4 mm in diameter and 50 mm in height, creating the void in the physical phantom where the dosimeters are inserted. A total of 51 cylinders were placed over the phantom and the position of each cylinder is shown in Supplemental Table 1. Bones, upper and lower respiratory tract, liver, kidneys, uterus, and fetus are present in the physical phantom representation, and the remaining structures were omitted. The reason for this is that the phantom is intended to be open‐source, that is, freely available to other research groups for manufacturing, so simplicity and reproducibility of the phantom are imperative. The contours of the parenchymal organs (liver and kidneys) and fetus with an adjacent uterus were left for better orientation and visualization when the phantom was subjected to imaging diagnostics. For structures that will be filled with a soft tissue substitute material, holes are intentionally added so that the replacement material can flow into all parts of the mold seen in Supplemental Figure 1.

The entire phantom is divided into 18 horizontal layers. For the structures that span several layers and are cut into several parts, registration points and mounting plates/brackets are added to connect the outer shell of the mold and each part, holding them in the exact place (as also seen in Supplemental Figure 1).

Finally, the custom‐made bracket that will hold the physical phantom slices in the place was 3D modeled.

Two FDM/FFF (Fused Deposition Modelling/Fused Filament Fabrication) 3D printers with different plate sizes (i.e., 3D printing area) were used for the 3D printing process: a smaller Prusa i3 MK3 (Prusa Research; Prague, Czech Republic) and a larger Creality CR10‐S4 (Shenzhen Creality 3D Technology Co, Ltd.; Shenzhen, China).

The molds were printed with PLA (“*polylactic acid*”) filament with a mass density of 1.24 g/cm^3^. Since it is one of the cheapest materials available on the market with a low glass transition temperature (around 54°C) it was quite easy to remove outer shells with a heat gun and discard the remains.

After the 3D printing, postprocessing of the molds was performed in terms of removing the support material. In case of wall breakage, parts were joined with cyanoacrylate glue (“super glue”) and additionally strengthened with a 3D pen using PLA plastics. All larger defects (holes) that occurred during 3D printing were also corrected and closed with the same material using a 3D pen.

Polycarbonate (PC) filament was used for the 3D printing of the threaded part of the phantom holder, which is characterized by high strength and stiffness.^36^ Instead of PC filament, any other material characterized by high strength could be used for 3D printing of this part that will have to endure high forces.

#### 2.1.2 Tissue substitutions

To make the process of physical phantom creation reproducible, it was decided to produce the phantom from three different tissue substitute materials with similar properties as published in previous research.^37^ For molding the phantom, materials currently available on the market were used, which led to using of different component ratios than in the above‐mentioned work.

A two‐component mixture of polyurethane (PU) rubber (VytaFlex 60, Smooth‐On, Inc.; Macungie, Pennsylvania) and CaCO_3_ in the ratio of 97.2% PU + 2.8% CaCO_3_ is used for soft tissue substitution (STS). Lung tissue substitution (LTS) is created as a mixture of soft tissue substitution (92.6%) and polystyrene (Styrofoam) balls (7.4%) with a diameter of 2–3 mm, which is further crushed with a food processor blender. When blending polystyrene balls, it is important to mix them with water to prevent melting or self‐ignition, and fully dry them before mixing them with a soft tissue substitution mixture. A mixture of epoxy resin (EpoxAcast 690, Smooth‐On, Inc.; Macungie, Pennsylvania), SiO_2_ and CaCO_3_ in mass ratios of 65%, 5% and 30% is used to make bone tissue substitutes (BTS). Good sifting of the CaCO_3_ powder is necessary for preventing the formation of lumps in the mixture.

Casted cubes used for measurements, together with their CT scans are seen in Supplemental Figure 2. The attenuation values of substitute tissues are summed in Table 1 and correlate with the average attenuation coefficients and density of the corresponding tissues in the human body.

**TABLE 1:** The comparison of the measured substitute tissues attenuation values and mass density in Tena phantom to corresponding tissue of the human body from references.

Tissue	CT attenuation value (HU)	Mass density (g/cm^3^) ^a^
Soft tissue	−100 to 70^b^	1.02
Lung	−900 to −400^b^	0.26
Bone	300 to 1000^b^	1.55

Tissue substitute	CT attenuation value ± SD (HU)	Mass density (g/cm^3^)
Soft tissue—STS^c^	30 ± 5	1.03
Lung—LTS	−750 ± 80	0.287
Bone—BTS^d^	450 ± 45	1.274

Abbreviations: BTS, Bone tissue substitute; HU, Hounsfield units; LTS, Lung tissue substitute; SD, Standard deviation; STS, Soft tissue substitute.

^a^ Scott et al^51^

^b^ Kalender, 2011^55^

^c^ The attenuation coefficients of the replacement soft tissue of 30 HU were obtained as the average value of the attenuation values of the muscle tissue, fat tissue, blood and other soft tissues.

^d^ The attenuation values of the substitute bone tissue of 440 HU correspond to the average value of the cortex and medulla of the bone.

### 2.2 Physical phantom generation

Before the substitution material was poured into the molds, small imperfections that may have remained were sealed using a clear varnish or sealant to prevent leaking of the substitute tissue components through small imperfections.

First, the lung tissue substitute was cast in the molds because it is the most viscous material of the three. Manual compression ensured that we filled all the hard‐to‐reach places. The Lung tissue substitute had to cure for 24 h.

**FIGURE 1:**
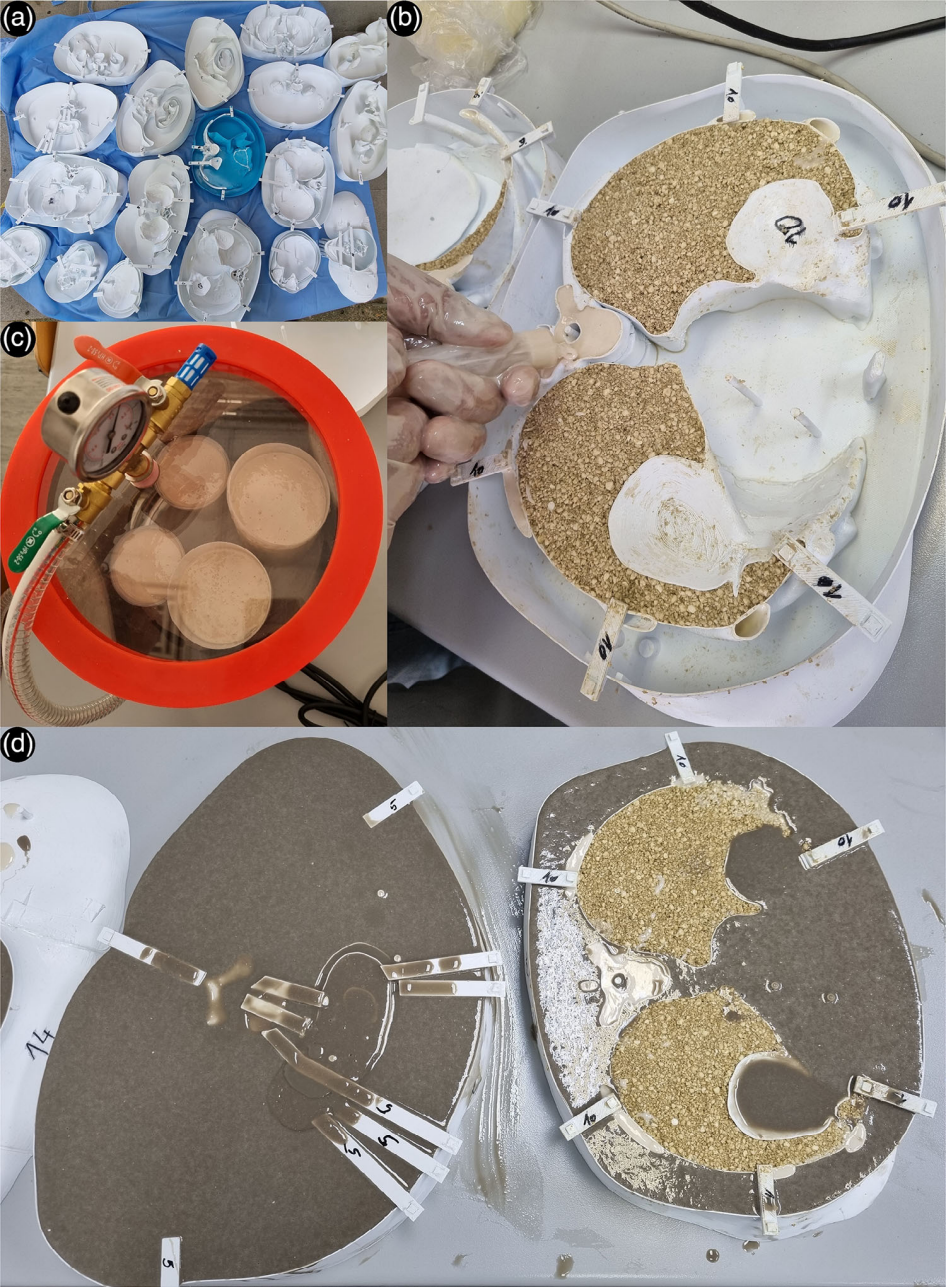
The process of physical phantom generation. (a) Molds before casting. (b) Degassing of the bone tissue substitution using a vacuum chamber. (c) Casting of the bone tissue substitution using syringes with already casted lung tissue substitute visible in corresponding compartments. (d) The casting of the soft tissue substitution and resting for polymerization.

The bone tissue substitute was then cast into the molds. Because of its short pot life, the mixing of a small amount of substitution material, and casting was repeated several times to prevent polymerization and difficult casting. Before casting, the mixture was placed in a vacuum chamber to prevent the accumulation of gas bubbles and to improve the homogeneity of the substitution tissue (Figure 1b). The alternative could be to shake the casted molds using a concrete vibrator or something similar. Due to its viscosity and because most bone molds are narrow, the bone substitute tissue was injected using syringes (Figure 1c).

Finally, after pouring the bone substitute material, the soft tissue substitute, as the least viscous material, was poured filling the rest of the available space in each mold (Figure 1d). Same as the bone tissue substitution, before pouring the soft tissue substitution, the mixture was de‐gassed to prevent gas bubble accumulation and for better homogeneity. This process was performed in two steps for the entire phantom, also due to the PU's short pot life. The same soft tissue substitute is used to fill molds that produce alignment pins placed on each side of the layers to align all 18 layers of the physical phantom. It is worth noting that fetus was molded as STS in full.

Once fully cured, the outer shell of the molds and the mounts for the moving parts were easily removed using an industrial hot air gun. Before assembling the physical phantom, the excess material from the upper margin of each slice was sanded to achieve a flat surface and better matching of the layers.

**FIGURE 2:**
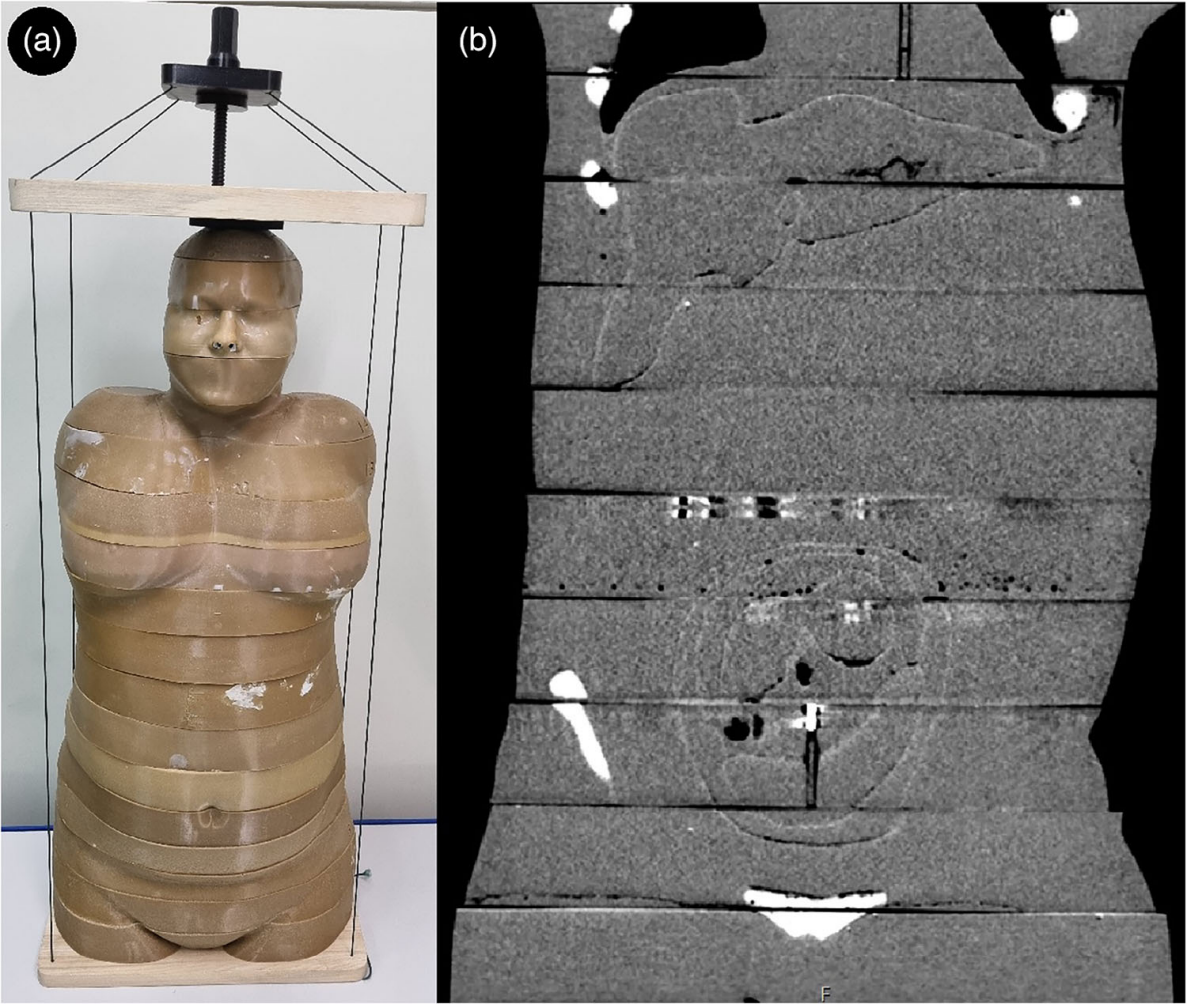
The finished physical phantom. (a) Depiction of the phantom layers being held together by custom‐made brackets and (b) display of the physical phantom's abdomen and pelvis on the CT scan.

The post‐processed slices were arranged according to the numbers between custom–made brackets consisting of two flat, wooden plates that were previously machined using a CNC machine according to the matching 3D model and 3D printed head holder with thread as seen in Figure 2a. Soft tissue substitute alignment pins were inserted into the matching holes, adding stabilization between the slices.

### 2.3 Radiotherapy plan

For the model verification, 3D conformational radiotherapy (RT) treatment plan was used as described previously.^25^ Virtual Tena phantom was used as the calculation dataset. The planning target volume (PTV) was defined according to the clinical recommendations for the breast treatment, as well as organs of risk (OAR): lung left, lung right, heart, medulla and larynx. One of the standard fractionation (50 Gy in 25 fractions) for breast radiation therapy was used. The recommendations for the PTV were to be covered with doses in the range 95%–107% of the prescription dose, maximum dose *D*_max_ ≤ 110% and mean dose ± 1% of the prescription dose.

The radiotherapy treatment plan was obtained using commercial planning software Varian Eclipse 15.6 (Varian Medical Systems Inc., Palo Alto, USA), and was made for linear accelerator Siemens Oncor Expression, using only 6 MV photon beams. The plan consists of two main opposite fields in which beams are tangential to the chest wall, and every beam has an additional small field (patch field) from the same direction. To guarantee a more homogeneous dose in the PTV, an additional fifth field (posterior—anterior direction) was added (Supplemental Figure 3).

Prior to the irradiation, we set up the phantom on the couch of the treatment machine with the head of the phantom towards the gantry, in the supine position. The linear accelerator is equipped with the imaging guidance system (Optivue Siemens Medical Solutions) used for 6 MV megavoltage planar imaging, and we use it to check the position of the patients and phantoms in the anterior‐posterior and mediolateral direction.

### 2.4 Computational methods

MC simulations of the radiotherapy plan were performed using Monte Carlo N‐Particle transport code version 6.2.^38^ The model of the accelerator, as well as the voxelized Tena phantom setup, are described in previous publications.^25^, ^28^, ^39^, ^40^, ^41^ The phantom materials used in MC simulations were as described in the previous section. Exact elements and weight fractions are given in Supplemental Table 2. Though materials used for simulation had similar attenuation (in terms of HU units) and physical density as ICRU materials, we did simple verification of the materials’ radiation scattering properties using simplified irradiation geometry. Namely, the source was defined as isotropic with photon spectra obtained from previous simulations.^25^ Box with dimensions of 30 × 30 × 30 cm^3^ were placed 90 cm from the isotropic source and filled with smaller boxes 5 × 5 × 0.2 cm^3^ in the center of the box (where the depth was 0.2 cm). In this way, 150 detectors were used to record the percentage depth doses (PDDs) for each material. Both box and detectors were filled with appropriate material composition for soft tissue, lung and bone from ICRP,^42^ ICRU^43^ and our newly developed compositions. 3 × 10^9^ particles were simulated in each simulation and a F6 tally (for recording energy deposited in the cell) was used to calculate deposited energy. The PDDs of ICRU, ICRP and our materials were then compared. In the simulations with the Tena voxelized phantom only ICRP and our newly developed substitute materials were used. Absorbed doses were calculated using +F6 tallies that are positioned in each measuring point as described in the phantom development section. Tallies were positioned in spheres with diameters matching the voids for dosimeters in physical phantom and overlapped with voxelized phantom geometry. DXTRAN sphere with a 10 cm radius was set in the fetus’ head to speed up the calculations. The spectra were collected in all measuring points (using an F5 tally which collects particle flux at defined points). The radiotherapy plan consisted of five different fields, as described in previous publication^25^ and previous sections of this paper, and all five fields were simulated separately in MCNP.

### 2.5 Experimental measurements

Absorbed doses in the phantom were measured with the radio photoluminescent (RPL) dosimeters type GD‐352 M manufactured by ATCG, Japan. The plastic holder of the GD‐352 M dosimeters has a filter for energy‐dependence compensation.^44^ Measurements of the RPL signals were carried out with the FDG‐1000 reader.^45^ RPL dosimeter is based on the RPL phenomenon of silver‐activated phosphate glass exposed to ionizing radiation, that is, RPL centers are formed in the glass after irradiation. In the reader, an ultraviolet (UV) beam is used to excite RPL centers. Emitted RPL light is collected in the photomultiplier and the final signal, that is, proportional to the absorbed dose, is formed. The calibration of dosimeters was performed in a ^137^Cs gamma‐ray field in the Secondary Standard Dosimetry Laboratory at Ruđer Bošković Institute^46^ in terms of kerma “free in air” and conversion to the absorbed dose to water was performed using experimentally determined conversion factor (*D*_w_/K_air_ = 1.12).^47^ The combined uncertainty (k = 1) including reproducibility, calibration and angular dependence was 2.1% (for 1 mGy–2 Gy) and 2.7% (below 1 mGy). Detailed characteristics and principles of the used RPL dosimetry system can be found in the paper by Knežević et al.^48^

Dosimeters were placed in 49 positions within the fetus and structures in the rest of the phantom (Supplemental Table 3). Measured doses, *D*_w_ (mGy), are expressed as absorbed dose to water. All results are normalized at mean target dose and expressed in mGy/Gy.

## 3 RESULTS

### 3.1 Physical phantom development

The finished physical phantom Tena is 88.9 cm in height, and its mass is 49.7 kg without a bracket. It is divided into 18 horizontal layers: 17 layers of 50 mm and one layer of 39.9 mm height as seen in Figure 2. It consists of three types of substitution tissues: STS, BTS and LTS. Physical and radiological properties of tissue substitutions are summarized in Table 1.

### 3.2 Monte Carlo simulations

**FIGURE 3:**
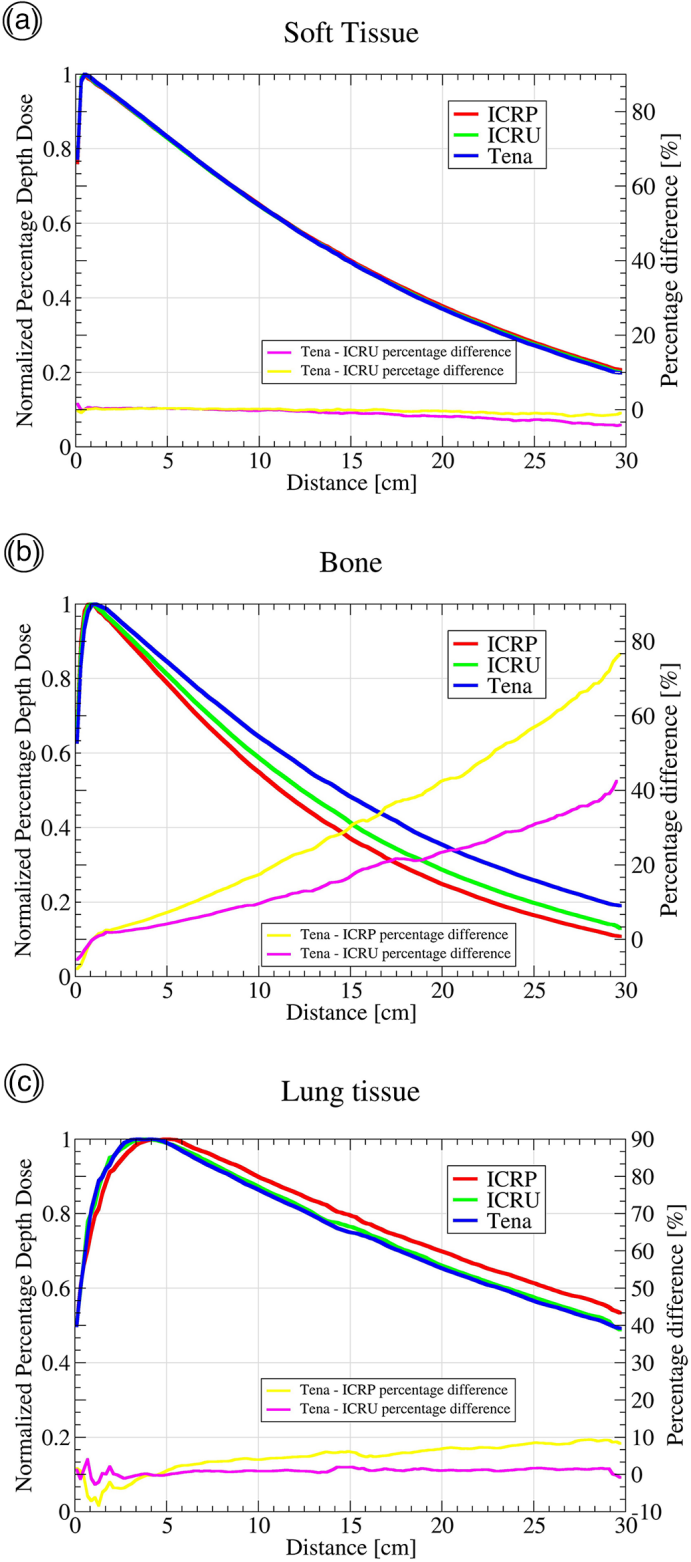
Percentage depth doses for different types of materials obtained by MC simulations. The red line represents ICRP materials, green ICRU materials and blue line materials used in this study for the construction of a physical representation of the anthropomorphic phantom (named Tena). The percentage difference between Tena and ICRP materials is shown in the yellow line and the percentage difference between Tena and ICRU materials is shown in the pink line (a) soft tissue; (b) bone; (c) lung.

To assess PDD for each material, MC simulations for the cube filled with each material, defined in three different ways (using ICRP material, ICRU material, and the Tena materials), were performed. PDDs are normalized to their maximum value and the results are shown in Figure 3. For STS and LTS, and their ICRU and ICRP counterparts' differences in PDDs are within 5% at the depth of 20 cm, but higher for BTS. Nevertheless, radiation passes only several centimeters of bones in the body where differences in PDD values are within 5% (in the first 4 cm of the BTS cube).

### 3.3 Experimental measurements

All measurements normalized per Gy of mean target dose are presented in Supplemental Table 3.

For the computational determination of organ dose, two sets of MC simulations were performed. In the first one, ICRP materials were used, and in the second, materials which were developed within this study. Results for the chosen position of both simulations are compared with experimental results and shown in Table 2. The differences between experimental and computational results are within 20% for Tena phantom materials. It is worth noting that TPS calculated fetal dose was below 15 mGy, while fetal dose estimated according to the AAPM 36 and 158 was between 30 mGy and 60 mGy.

**TABLE 2:** Comparison of MC simulations and experimental results of doses received by the radiotherapy plan.

	Absorbed dose [mGy/Gy]			Differences [%]	
	MC simulation		Experiment		
	ICRP materials	Tena materials	Measurements in phantom	Experiment/ICRP MC simulation	Experiment/Tena MC simulation
Right iliac bone	0.97 (15%)	0.81 (9%)	0.74	‐23.7	‐8.6
Placenta	1.10 (19%)	0.91 (8%)	0.87	‐20.9	‐4.4
Fetus, body	1.38 (13%)	1.29 (8%)	1.36	‐1.4	5.4
Fetus, head	1.37 (9%)	1.70 (11%)	1.48	8.0	‐12.9
Left breast, LIQ	957.91 (5%)	943.71 (4%)	1048	9.6	11.1
Left breast, LOQ	1000 (6%)	1000 (6%)	1000	–	–
Right thyroid lobe	9.46 (31%)	11.94 (23%)	10	5.7	‐16.2

*Note*: The relative errors of MC simulations are given in brackets. The relative uncertainty of dosimetry measurements is 2.1%.

## 4 DISCUSSION

In this study, a physical phantom of the pregnant female, in her 18^th^ week of pregnancy, was developed (Figure 2a). Our previously published computational Tena phantom was used^25^ as a template and organ's .STL files were used to generate molds for casting. Three different substitute tissues: STS—soft tissue substitution, BTS—bone tissue substitution and LTS—lung tissue substitution, that are suitable for application in photon MV fetal dosimetry, were cast in these molds.

The molds for the Tena phantom were 3D printed on two different FDM/FFF—based 3D printers using affordable PLA (lactic acid polymer) material. Due to its physical properties (mass density, ρ = 1.20 g/cm^3^), radiological properties (CT number 50–300 HU) and chemical composition, PLA material has good characteristics in simulating soft tissues and spongious bone.^49^, ^50^

The choice of substitute tissues for the phantom production was roughly based according to a published article by Winslow et al. but with materials that are currently available on the market.^37^ This has led to the use of different component ratios than those used in the work of Winslow et al., but similar to what was reported by Hoerner et al. in their work.^21^, ^37^ Compared to Hoerner et al. who used VytaFlex 40, Smooth‐On polyurethane rubber was used in our soft tissue substitutes. Thus our mixture consists of VytaFlex 60, Smooth‐On PU rubber with a higher shore hardness of 60A, compared to 40A, ensuring the longevity of the phantom due to wear resistance during use and manipulation. The STS mass density is 1.03 g/cm^3^ and corresponds well to a human body soft tissue with a mass density of 1.02 g/cm^3^. CT attenuation of the STS, obtained as the average value of the attenuation values of the muscle tissue, fat tissue, blood, and other soft tissues, is 30 ± 5 HU, comparable to Hoerner et al. who reported 0–20 HU (average 10 HU) CT number for soft tissue substitute material, while CT number of the human soft tissue is −100 to 70 HU (Table 1). The BTS was made as a homogenous mixture of cortical and medullar bone, and it was made as epoxy resin (EpoxAcast 690, Smooth‐On), SiO2, and CaCO3 mixture, in mass ratios of 65%, 5%, and 30% while Hoerner et al. mixed fiberglass resin (Bondo, 3 M), SiO2 and CaCO3 in mass ratios of 51.0%, 25.5%, and 3.5%. The mass density of the BTS is 1.274 g/cm^3^, compared to human bone tissue with 1.55 g/cm^3^. CT number of BTS, corresponding to the average value of the cortex and medulla of the bone, was 453 ± 59 HU, compared to Hoerner et al. who accepted CT values ranging from 650 to 810 HU (mean 725 HU), while human bone tissue has CT number 300–1000 HU.

The mass density of the LTS is 0.287 g/cm^3^ and the CT number is −750 ± 80 HU, compared to human lung tissue with a mass density of 0.26 g/cm^3^ and a CT number of −900 to −400 HU.

The most similar physical phantom of a pregnant female, of several produced and mentioned in the introduction section, is the one developed by Hoerner et al..^21^ That phantom consists only of the abdomen and pelvis, and it does not allow experimental determination of the fetal dose in real conditions, while our phantom allows simulating radiotherapy of various pathologies, from head and neck tumors to the mediastinum, etc. Phantom developed by Shirkhani et al. consists of the thorax, abdomen and pelvis region also, but it is made of different materials, paraffin and cork.^22^ Phantom reported by Matsunaga et al. is created in such manner that “pregnant belly” made of polyurethane resin could be mounted on Alderson RANDO phantom.^23^ The properties of different physical phantoms currently available are summarized in Supplemental Table 4.

The MC simulations of all three materials (ICRP, ICRU and Tena), PDDs for STS do not differ more than 5% up to a depth of 25 cm (Figure 3), while for BTS and LTS discrepancy is higher. The Tena material composition slightly overestimates the doses in the BTS for higher depths, while for LTS Tena PDD curve fits almost perfectly with the ICRU PDD curve.  It is worth noting that ICRU material composition is based on real tissues, while both the Tena composition and the ICRP are artificial materials^51^​. Tena tissue substitutes are sufficient for creating the physical representation of the phantom, especially since radiation does not usually pass through bones thicker than 4 cm.

According to the AAPM 36 and 158, the determination of the fetal doses during radiotherapy should be improved by performing MC simulations and measurements in dedicated anthropomorphic phantoms.^14^, ^15^ This was done as a part of our research using both experimental and computational approaches. In other words, MC methods can provide information such as spectral or angular distribution or even the dose to the whole organ, while experimental methods can provide reliable data for complex RT plan in a short time. Nevertheless, in this work, measurements are considered as a golden standard since MC simulations in distant areas from the field edge were performed and 10% uncertainty was accepted. However, the best approach is when experiment and simulations are combined as stated above.

Experimental results shown in Table 2 and Supplemental Table 3 clearly indicate that for the whole radiotherapy plan of 50 Gy fetus head is going to receive 74.13 mGy, which is just above the threshold of 50 mGy^8^, ^52^ This is in line with the results of MC simulations where a dose of 84.9 mGy was calculated for the dosimeters. positioned in the fetus head. Additionally, we estimated fetal dose using the AAPM guidelines.^14^, ^15^ Although this is a rough estimate, with fetal dose between 30 mGy and 60 mGy, the results were comparable to ours. A dose to the fetus head could be even lower if the fetus was in headfirst position in uterus as fetuses in the first two months are freely movable in the uterus. Although there is a 14% difference between the experimental and computational results, caused by measurement uncertainty and MC statistical error, our newly developed low‐cost anthropomorphic phantom proved to be reliable in experimental determination of fetal dose. Though TPS does not estimate the dose to the fetus accurately because it is far from the filed edge,^31^, ^53^, ^54^ we calculated it in the measuring point. It was found to be below 15 mGy which is in line with previous findings that TPS underestimates the doses far from the field.^31^, ^53^, ^54^

Comparison of the experimentally obtained fetal dosimetry results with the Tena phantom was only possible with the data published by Shirkani et al., given that the mentioned group was the only one to perform dosimetry measurements for breast radiotherapy, while the other groups measured the radiation dose during imaging diagnostics. In the aforementioned work, the fetal dose in the second trimester, that is, 24th week of pregnancy was 140–190 mGy.

The cost of the material for the generation of the Tena physical phantom is about €1000, which is less than any commercial phantom and makes it affordable to a wide scientific audience. The whole process of phantom creation takes several weeks of intermittent work. In the near future we intend to benchmark this phantom for use in diagnostic and interventional radiology fetal dosimetry as well as in proton therapy.

## 5 CONCLUSIONS

In this study, a physical phantom of a pregnant woman at 18 weeks of pregnancy was developed, and to date, it is the first available physical phantom of a pregnant woman. The whole process of phantom creation takes several weeks of intermittent work, and the physical phantom can be manufactured with low expenses—around €1,000.

Both, the calculated and experimentally determined fetal doses during breast radiotherapy agree within 14% (85 mGy for computational dosimetry and 74 mGy for experimental dosimetry) which validates the use of the Tena phantom developed in this study for the fetal dosimetry in MV photon radiotherapy.

Materials used for developing a physical representation of the phantom showed reliability since PDD does not differ more than 5% for STS (at 25 cm depth), 10% for LTS (at 20 cm depth) and 5% for BTS (at 4 cm depth) from the values determined for ICRU materials.

## AUTHOR CONTRIBUTIONS

VK – 3D printing of the molds, preparing casting materials, casting, performing CT scans, writing the paper; HB—preparing the manuscript, preforming MC simulations; MK, AI—preparing the radiotherapy plan, casting; ŽK, MM, MN—performing the experimental measurements; TT—performing CT scans, concepting the manuscript; DF—conceptual idea of the paper, writing the paper

## CONFLICT OF INTEREST STATEMENT

The authors declare no conflicts of interest.

## Supporting information

Supplemental Information.

## Data Availability

The data that support the findings of this study are available from the corresponding author upon reasonable request.
