# Supplementary material for: Development and validation of the low‐cost pregnant female physical phantom for fetal dosimetry in MV photon radiotherapy
Source: J Appl Clin Med Phys. 2023 Dec 27;25(2):e14240. doi: 10.1002/acm2.14240 (PMC10860449; doi:10.1002/acm2.14240)
Supplement: Supplementary file 1 — Supplemental Information. [file ACM2-25-e14240-s001.docx]

**Supplementary material**

“Ready to 3D print” STL files of the molds are available in our previously published paper.


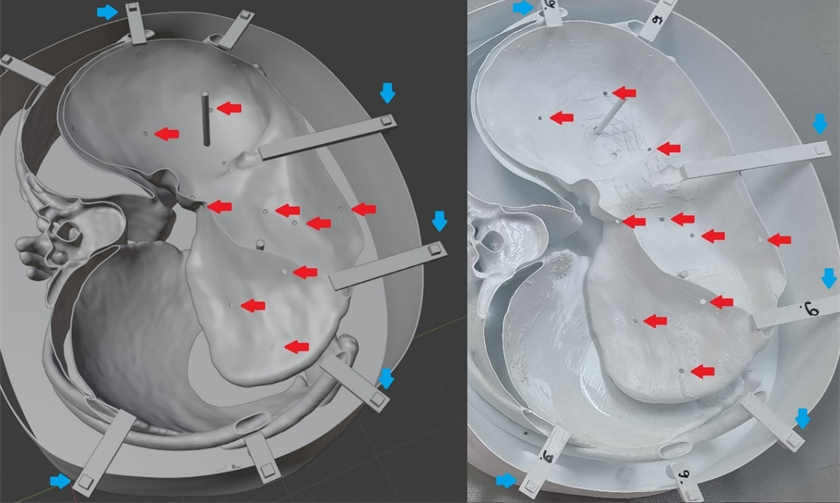


Supplemental Figure 1. CAD 3D model of the one mold on the left and the same mold 3D printed on the right. Red arrows depict intentionally generated holes for better flow of the soft tissue substitution throughout the mold. Blue arrows depict registration points and attachment plates/brackets holding mold parts in the exact place. The wall thickness of structures (compartments) inside the molds is 0,6 mm, and the thickness of the mold’s outer wall is 0,8 mm. That wall thickness is sufficient to create thick enough contours to make the structure's shells solid without affecting the quality of the 3D print in terms of artifacts (i.e., holes) after the 3D printing process, and visibility in CT scanning as seen in Figure 2.


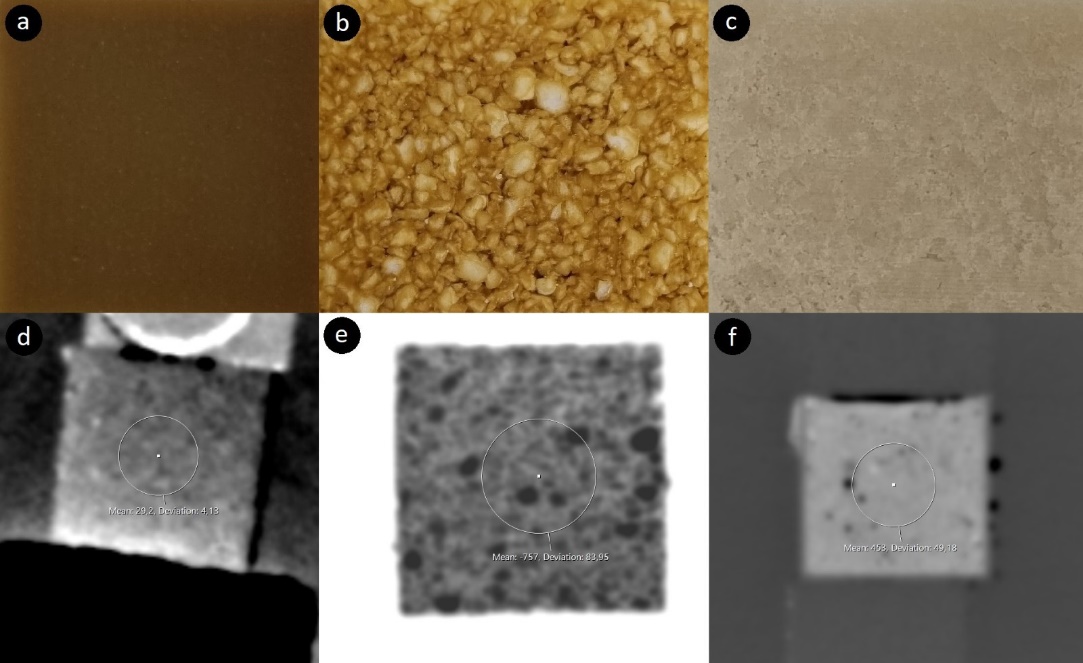


Supplemental Figure 2. The actual appearance of cast test cubes: a. the soft tissue substitution material, b. the lung tissue substitution material and c. The bone tissue substitution material. The appearance of the casted test cube scanned with a CT scanner: d. CT scan of the soft tissue substitution cube e. CT scan of the lung tissue substitution cube, f. CT scan of the bone tissue substitution cube.


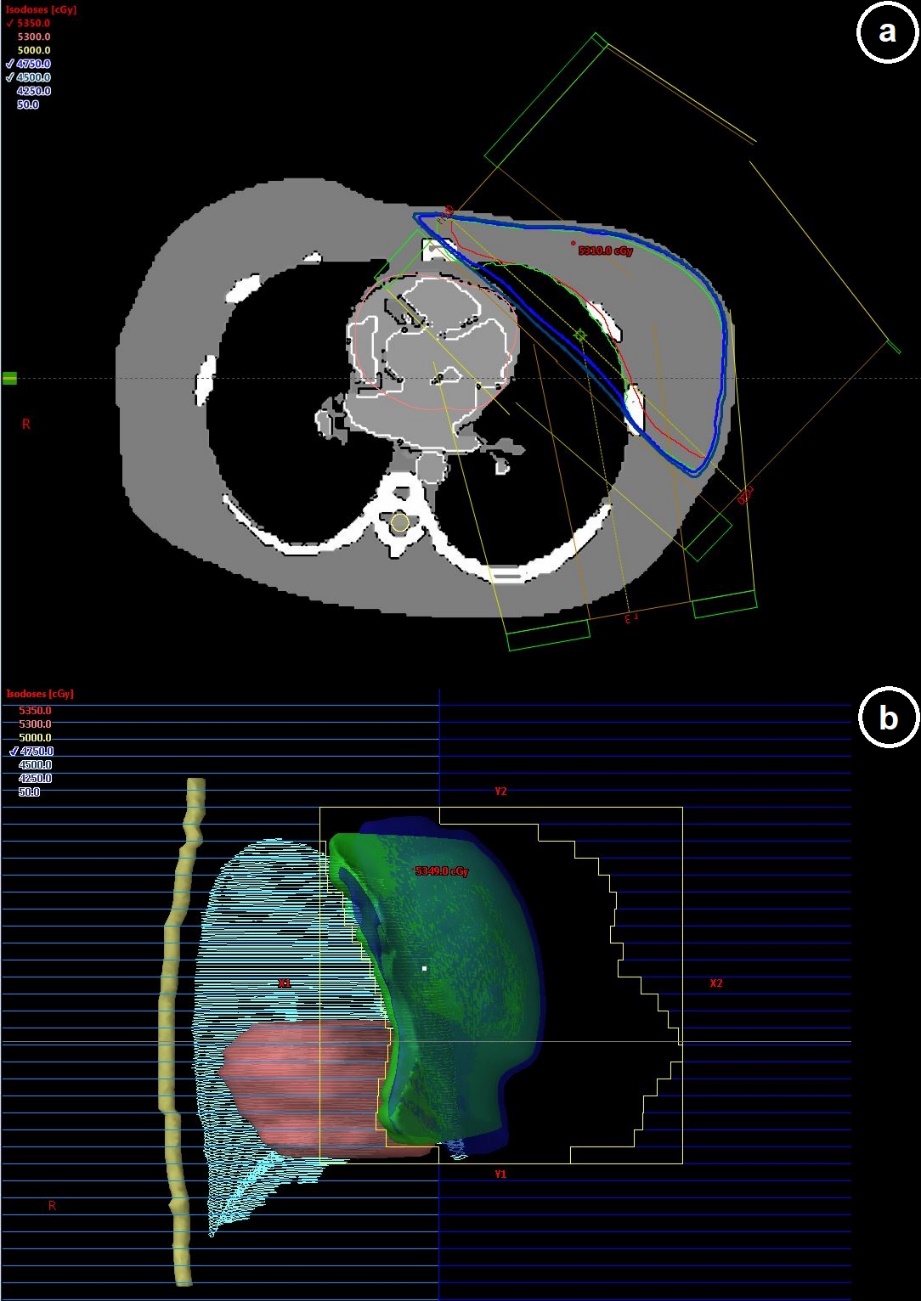


Supplemental Figure 3. The radiotherapy plan for left breast. a. beam arrangements in the Eclipse treatment planning software showing two main fields and three “patch” fields as well as organ lines (PTV and OAR) and isodose lines . b. Beam Eye View of the F1 main field with depicted PTV and OAR.

Supplemental Table 1. A list of all 51 dosimeter placement sites in relation to anatomical structures. LOQ – lower outer quadrant, LIQ – lower inner quadrant, UOQ – upper outer quadrant, UIQ – upper inner quadrant.

| Slice No. | Dosimeter No. | Structure | Position in the slice (Lower /Upper half)^a^ |
| --- | --- | --- | --- |
| 1 | 0 | / | / |
| 2 | 0 | / | / |
| 3 | 3.1. | Right iliac bone | L/U |
|  | 3.2. | Left iliac bone | L/U |
|  | 3.3. | Rectum | L |
|  | 3.5. | Placenta | L |
| 4 | 4.1. | Fetus, body | L |
|  | 4.2. | Fetus, head | U |
| 5 | 5.1. | Ascending colon | L/U |
|  | 5.2. | Terminal ileum | U |
|  | 5.3. | Above fetal head | L |
| 6 | 6.1. | Right kidney, caudal pole | U |
|  | 6.2. | Left kidney, caudal pole | U |
|  | 6.3. | Descending colon | L/U |
| 7 | 7.1. | Liver, right lobe (caudal segments) | L/U |
|  | 7.2. | Duodenum | L/U |
|  | 7.3. | Right kidney, cranial pole | U |
|  | 7.4. | Lumbar spine (spinal cord) | U |
|  | 7.5. | Pancreas/jejunum | U |
| 8 | 8.1. | Left kidney, cranial pole | L |
|  | 8.2. | Stomach | L/U |
| 9 | 9.1. | Liver, right lobe (cranial segments) | L/U |
|  | 9.2. | Liver, left lobe | L/U |
| 10 | 10.1. | Thoracic spine (spinal cord) | L |
|  | 10.2. | Right lung, lower lobe | L |
|  | 10.3. | Left lung, lower lobe | L |
|  | 10.4. | Heart, LAD coronary artery | L |
|  | 10.5. | Heart, LM coronary artery | U |
| 11 | 11.1. | Right lung, middle lobe | L |
|  | 11.2. | Left lung, upper lobe (lingula) | L |
|  | 11.3. | Right breast, LOQ | L |
|  | 11.4. | Right breast, LIQ | L |
|  | 11.5. | Sternum | L |
|  | 11.6. | Left breast, LIQ | L |
|  | 11.7. | Left breast, LOQ | L |
| 12 | 12.1. | Right breast, UOQ | U |
|  | 12.2. | Right breast, UIQ | U |
|  | 12.3. | Left breast, UIQ | U |
|  | 12.4. | Left breast, UOQ | U |
|  | 12.5. | Right lung, superior lobe | U |
|  | 12.6. | Left lung, superior lobe | U |
|  | 12.7. | Esophagus, middle third | L/U |
| 13 | 0 | / | / |
| 14 | 14.1. | Cervical spine (spinal cord) | L/U |
|  | 14.2. | Right thyroid lobe | L |
|  | 14.3. | Left thyroid lobe | L |
|  | 14.4. | Right humerus head | L |
|  | 14.5 | Left humerus head | L |
| 15 | 15.1. | Right parotid gland | U |
|  | 15.2. | Left parotid gland | U |
| 16 | 16.1. | Right eye, lens | U |
|  | 16.2. | Left eye, lens | U |
| 17 | 17.1. | Brain, right lobe | L/U |
|  | 17.2. | Brain, left lobe | L/U |

^a^ L – lower / U – upper half

Supplemental Table 2. Elements and the appropriate weight fractions for each material of physical phantom representation used in MC simulations.

|  | Weight fractions | | |
| --- | --- | --- | --- |
| Element | Lung   (ρ=0.287 g/cm^3^) | Bone    (ρ=1.274 g/cm^3^) | Soft tissue   (ρ=1.03 g/cm^3^) |
| H | 0.07 | 0.05 | 0.07 |
| C | 0.71 | 0.5 | 0.69 |
| N | 0.05 | 0.04 | 0.05 |
| O | 0.16 | 0.28 | 0.18 |
| Si | - | 0.02 | - |
| Ca | 0.01 | 0.12 | 0.01 |

Supplemental Table 3**.** Results of experimental measurements. Measurements from dosimeters placed in each humerus head were omitted due to technical reasons. Dose D_w_ (mGy/Gy) – normalized dose. (LOQ – lower outer quadrant; LIQ – lower inner quadrant; UOQ – upper outer quadrant; UIQ – upper inner quadrant).

| Slice No. | Dosi- meter | Structure | Position in  the slice^a^ | | Dose Dw, mGy/Gy | Slice No. | Dosi- meter | Structure | Position in the slice^a^ | | Dose Dw, mGy/Gy |
| --- | --- | --- | --- | --- | --- | --- | --- | --- | --- | --- | --- |
| 3 | 3.1. | Right iliac bone | | L/U | 0.74 | 11 | 11.1. | Right lung, middle lobe | | L | 18.27 |
|  | 3.2. | Left iliac bone | | L/U | 0.89 |  | 11.2. | Left lung, upper lobe (lingula) | | L | 231.35 |
|  | 3.3. | Rectum | | L | 0.81 |  | 11.3. | Right breast, LOQ | | L | 21.01 |
|  | 3.5. | Placenta | | L | 0.87 |  | 11.4. | Right breast, LIQ | | L | 30.56 |
| 4 | 4.1. | Fetus, body | | L | 1.36 |  | 11.5. | Sternum | | L | 90.86 |
|  | 4.2. | Fetus, head | | U | 1.48 |  | 11.6. | Left breast, LIQ | | L | 1047.77 |
| 5 | 5.1. | Ascending colon | | L/U | 1.74 |  | 11.7. | Left breast, LOQ* | | L | 1000.00 |
|  | 5.2. | Terminal ileum | | U | 2.07 | 12 | 12.1. | Right breast, UOQ | | U | 14.63 |
|  | 5.3. | Above fetal head | | L | 2.52 |  | 12.2. | Right breast, UIQ | | U | 26.78 |
| 6 | 6.1. | Right kidney, caudal pole | | U | 2.80 |  | 12.3. | Left breast, UIQ | | U | 994.97 |
|  | 6.2. | Left kidney, caudal pole | | U | 3.80 |  | 12.4. | Left breast, UOQ | | U | 1002.01 |
|  | 6.3. | Descending colon | | L/U | 4.63 |  | 12.5. | Right lung, superior lobe | | U | 19.58 |
| 7 | 7.1. | Liver, right lobe (caudal segments) | | L/U | 3.90 |  | 12.6. | Left lung, superior lobe | | U | 157.08 |
|  | 7.2. | Duodenum | | L/U | 4.81 |  | 12.7. | Oesophagus, middle third | | L/U | 35.01 |
|  | 7.3. | Right kidney, cranial pole | | U | 3.92 | 14 | 14.1. | Cervical spine (spinal cord) | | L/U | 9.08 |
|  | 7.4. | Lumbar spine (spinal cord) | | U | 4.36 |  | 14.2. | Right thyroid lobe | | L | 10.06 |
|  | 7.5. | Pancreas/jejunum | | U | 6.78 |  | 14.3. | Left thyroid lobe | | L | 13.53 |
| 8 | 8.1. | Left kidney, cranial pole | | L | 21.29 | 15 | 15.1. | Right parotid gland | | U | 5.65 |
|  | 8.2. | Stomach | | L/U | 10.50 |  | 15.2. | Left parotid gland | | U | 8.44 |
| 9 | 9.1. | Liver, right lobe (cranial segments) | | L/U | 15.00 | 16 | 16.1. | Right eye, lens | | U | 7.65 |
|  | 9.2. | Liver, left lobe | | L/U | 52.05 |  | 16.2. | Left eye, lens | | U | 6.45 |
| 10 | 10.1. | Thoracic spine (spinal cord) | | L | 23.17 | 17 | 17.1. | Brain, right lobe | | L/U | 2.55 |
|  | 10.2. | Right lung, lower lobe | | L | 16.53 |  | 17.2. | Brain, left lobe | | L/U | 2.92 |
|  | 10.3. | Left lung, lower lobe | | L | 134.36 |  |  |  | |  |  |
|  | 10.4. | Heart, LAD coronary artery | | L | 46.80 |  |  |  | |  |  |
|  | 10.5. | Heart, LM coronary artery | | U | 83.98 |  |  |  | |  |  |

^a^ L – lower / U – upper half

Supplemental Table 4. Physical and radiological features of comparable physical phantoms. NA – not available, PU – polyurethane, Z_eff_ – effective nuclear charge.

| Phantom | Tena | Hoerner et al. | Shirkhani et al. | Matsunaga et al. | Rando woman | ATOM adult female |
| --- | --- | --- | --- | --- | --- | --- |
| Anatomical region | Head, neck, thorax, abdomen, pelvis, proximal thigs | Abdomen, pelvis | Thorax, abdomen, pelvis | Abdomen | Whole body | Whole body |
| Height (cm)/mass (kg) | 88.9/49.7 | NA | 60/NA | NA | 163/54 | 160/55 |
| Fetal age (weeks) | 17 | 15, 25, 38 | NA | NA | NA | NA |
| Substitution tissue | | | | | | |
| Soft tissue – material used | PU rubber, CaCO_3_ | PU rubber, urethane filler powder | Paraffin | PU resin |  |  |
| Density (g/cm^3^) | 1.03 | 1.041 | 1.01 | NA | 0.997 | 1.055 |
| CT number | 30 ± 5 | 0 – 20 | NA | NA |  |  |
| Z*_eff_* | 6.938 | NA | NA | NA | 7.6 | 7.15 |
| Bone tissue – material used | Epoxy resin, CaCO_3_, SiO_2_ | Fiberglass resin, CaCO_3_, SiO_2_ | Natural human bones | NA | Natural human bones |  |
| Density (g/cm^3^) | 1.274 | 1.60 | NA | NA |  | 1.6 |
| CT number | 450 ± 45 | 650 – 810 | NA | NA |  |  |
| Z*_eff_* | 10.775 | NA | NA | NA | NA | 11.5 |
| Lung tissue – material used | PU rubber, CaCO_3_, polystyrene balls | NA | cork | NA |  |  |
| Density (g/cm^3^) | 0.287 | NA | 0.3 | NA | 0.352 | 0.21 |
| CT number | -750 ± 80 | NA | NA | NA |  |  |
| Z*_eff_* | 6.897 | NA | NA | NA | 7.11 | 7.38 |
